# 

**Published:** 1817-04

**Authors:** 


### CORRESPONDENCE.

Dr. Johnson has to acknowledge the receipt of a letter, signed " A Well-wisher," and directed to him under frank. This anonymous Well-wisher has dissected the faults of the Medico-Chirurgical Journal with such candour, perhaps truth, that he is evidently a real friend to the work. Although it would be vain to hope to please all parties, yet there is something so sincere, so salutary, in the Well-wisher's letter, that Dr. J. solicits his real address, not from any motives of curiosity, but a desire to explain some points which cannot be done in a public Journal. That the Medico-Chirurgical Journal, with all its faults, has yet some share of professional respect in public estimation might be proved, if necessary, by the most convincing of all arguments, its *increasing circulation.* The Editors, however, are conscious of its errors and imperfections, and their course shall be marked by a constant effort at correction and improvement. On this account, the " Well-wisher's" letter is more acceptable than the most flattering eulogy, and the Medico-Chirurgical Journal will ever esteem his severest strictures as the most valuable communication it can receive.

Dr. Lee's very interesting case of Elephantiasis (accompanied by a Drawing) has been received, and will appear in our next. Mr. Fairburn's Case of Hydrocephalus; Hints on the Treatment of Cynanche Tonsillaris; Mr. Morrison's two cases of Death from eating inordinately of eggs, with the appearances on Dissection; and Mr. G. Nesse Hill's paper on " Contractions succeeding Ulcerations of the Skin," are received, and will have an early insertion.

The paper on *Tinctura Lyttæ* applied to a syphiloid ulcer, shall appear in our next *Porte-Feuille*, if possible. The able paper of Veridicus, respecting Dr. Spurzheim's cerebral dissections and demonstrations, is received, and shall meet attention. Dr. Yeates's case of Diseased Spleen, Dr. Mateos' Remarks on the degraded State of Medicine in Spain, are unavoidably deferred for want of room.

Mr. Charles Bell's third Report; Dr. Morrison's Cases of Tetanus; Dr. M'Lean on Endemic and Epidemic Diseases; and Transactions of the Medical Society of London, are under Review.

---

☞ Authors and Publishers are requested to send Copies of new Works for Review as early as possible after their publication. They will be reviewed early in proportion.

METEOROLOGICAL

